# Assessment of the localization of chondroitin sulfate in various types of endometrial carcinoma

**DOI:** 10.1371/journal.pone.0304420

**Published:** 2024-05-28

**Authors:** Sho Hosokawa, Yoshiaki Norimatsu, Akiko Shinagawa, Tetsuji Kurokawa, Yoshio Yoshida, Takeshi Nishikawa, Hisae Suzuki, Satoshi Irino, Tadao K. Kobayashi

**Affiliations:** 1 Department of Medical Technology, Faculty of Health Sciences, Ehime Prefectural University of Health Sciences, Ehime, Japan; 2 Department of Gynecology and Obstetrics, Faculty of Medical Sciences, University of Fukui, Fukui, Japan; 3 Department of Pathology, Nara Medical University Hospital, Nara, Japan; 4 Executive Director, Tenri University, Nara, Japan; Teikyo University, School of Medicine, JAPAN

## Abstract

**Introduction:**

This study aimed to assess the localization of chondroitin sulfate (CS), a primary extracellular matrix component, in the stromal region of endometrial carcinoma (EC).

**Methods:**

Immunostaining was performed on 26 endometrial endometrioid carcinoma (EEC) samples of different grades and 10 endometrial serous carcinoma (ESC) samples to evaluate CS localization. This was further confirmed by Alcian Blue (AB) staining as well.

**Results:**

In the G1-EEC samples, CS showed reactivity with fibrovascular stroma, supporting closely packed glandular crowding and papillary structures. As the grade increased, the original interstitial structure was re-established, and the localization of CS in the perigulandular region decreased. In the ESC samples, the thick fibrous strands supporting the papillary architecture showed reactivity with CS; however, the delicate stromal region branching into the narrow region showed poor reactivity. The AB staining results showed similar characteristics to the immunostaining ones.

**Conclusions:**

The characteristic localization of CS in various EC types was elucidated. The present study provides new information on endometrial stromal assessment.

## Introduction

Endometrial carcinoma (EC) is the most common malignant gynecological tumor in developed countries [1]. This type of tumor undergoes significant changes in its structure caused by excessive hormonal and other influences, and is further divided into two types based on its clinical and morphological characteristics [2]. Type I tumors are the most frequent, typically falling into the category of endometrial endometrioid carcinoma (EEC), and are composed of a proliferation of endometrial glands. This proliferation is often preceded by endometrial hyperplasia that is pathogenically linked to unopposed estrogen stimulation. By contrast, type II tumors, most often identified as endometrial serous carcinoma (ESC), are commonly described as estrogen-independent. These tumors typically present markedly different invasive patterns and prognoses from type I tumors. Their behaviors are not determined solely by their type, but are also affected by cross-talk between the cancer cells and the surrounding stroma [3].

The extracellular matrix (ECM) is a critical component of the tissue stroma that plays a supportive role in maintaining tissue morphology and is also involved in the environmental determinants of tumor cell behavior [4, 5]. The diversity in the morphological characteristics of fibrous cancer stromas and ECM components, as well as their clinical significance, have been recognized only recently [6]. Thus, it is important to examine tumor cells themselves and the surrounding environment—such as the stromal components and their localizations.

Chondroitin sulfate (CS) is a prominent component of the ECM and is abundant in the normal endometrial stroma throughout the menstrual cycle [7]. There is ample evidence for a characteristic CS localization and pro-tumorigenic role in the enhancement of cell proliferation, cell motility, and metastasis in several tumors—such as those of the breast, pancreas, and ovaries [8–10]. However, its behavior in the endometrial cancerous stromal compartment remains unclear.

In this study, the localization of CS in various types of ECs was assessed using immunostaining, and the characteristic localization could be easily detected by Alcian blue (AB; pH 1.0).

## Materials and methods

### Case selection and ethics statement

Thirty-six paraffin-embedded blocks containing endometrial tissue samples were acquired, which were collected from patients who had undergone hysterectomy at Fukui University Hospital between April 2018 and December 2020. They comprised 26 EEC (10 Grade 1 [G1], 6 Grade 2 [G2], and 10 Grade 3 [G3]) and 10 ESC, ranging in age from 49–75 years. Tumor subtypes were classified according to the World Health Organization system [11], and the tumors were graded according to the FIGO grading criteria [12]. This study was approved by the Institutional Review Board of Fukui University (approval no. 20200012) and was conducted in accordance with the principles of the World Medical Association’s Declaration of Helsinki. Written informed consent to publish this report and its accompanying images was obtained from all the patients who provided the tissue samples. The selection was conducted with appropriate informed consent and by accessing the hospital’s pathology archives. All data were accessed for research purposes between January 2021 and May 2021; however, no personally identifiable information was collected.

### Immunostaining

Immunostaining was performed to evaluate the localization of CS in the stromal regions of the ECs. Anti-CS mouse monoclonal antibody (CS-56) was purchased from Sigma-Aldrich Corp. (St. Louis, MO, USA) and used at a 1:1,000 dilution. To confirm the localization of vasculature in the stromal region, we also performed immunostaining for CD31, a vascular endothelial marker (ready-to-use clone JC70A; Agilent Technologies Denmark ApS, Glostrup, Denmark). First, 4 μm thick sections were cut from the formalin-fixed paraffin-embedded tissues, deparaffinized in xylene, and rehydrated in a graded series of ethanol dilutions. Endogenous peroxidase activity was blocked by incubating the sections in 3% hydrogen peroxide (H_2_O_2_) for 20 min at room temperature. All sample slides were washed in phosphate-buffered saline (PBS) before being exposed to the primary antibody overnight at 4°C. After washing, the sections were incubated for 30 min at room temperature with Histofine® Simple Stain™ MAX PO MULTI (Nichirei Bioscience Inc., Tokyo, Japan). Positive signals were visualized using a 3,3’-diaminobenzidine substrate kit (Nichirei Bioscience, Inc, Tokyo, Japan.). Tissue sections were counterstained with Mayer’s hematoxylin. A negative control experiment was performed using an antibody diluent that did not contain the primary antibody.

### Alcian Blue staining

The localization of CS in the stromal regions was also confirmed via AB staining (pH 1.0), which was mainly used to visualize sulfated mucosubstances. We cut 8 μm thick sections from the formalin-fixed paraffin-embedded tissues. These were deparaffinized in xylene and rehydrated in a series of ethanol dilutions with decreasing concentrations. Both slides were first incubated in 0.1 N hydrochloric acid for 1 min, then in 1% AB solution (pH 1.0) for 60 min at room temperature. Next, the slides were rinsed in 0.1 N hydrochloric acid, followed by running water. Finally, the sections were counterstained with Kernechtrot (Muto Pure Chemicals, Tokyo, Japan).

### Evaluation of staining

The evaluation of CS immunostaining in the stromal region surrounding the glands of G1 and G2-EEC was scored as follows: a) intensity: 0 = absent staining, 1 = weak staining, 2 = moderate staining, 3 = strong staining; b) distribution: 0 = absent staining, 1 = <25% of the focus circumference, 2 = 25–50% of the circumference, 3 = 50–75% of circumference, and 4 = >75% of circumference. The total score was calculated by adding the intensity and distribution scores for each region (which ranged between 0–7). The glandular architecture of the G3-EEC was extremely obscure and was therefore excluded from this evaluation. Next, for all of the EEC specimens, a 10× objective field was used, five fields were randomly selected, and the number of tumor nests surrounded by CS-positive stroma was counted. The staining evaluations were performed in consultation with three experienced researchers (S.H., Y.N., and T.N.). Tests to confirm statistically significant differences were conducted using Welch’s t-test or the Steel-Dwass test using “IBM SPSS Statistics 26” statistical software (IBM Japan, Ltd., Tokyo) or “R” statistical software (version 4.1.20; R Core Team 2021). Statistical significance was set at P < 0.05.

## Results

### Localization of chondroitin sulfate in EEC and ESC

**Fig 1:**
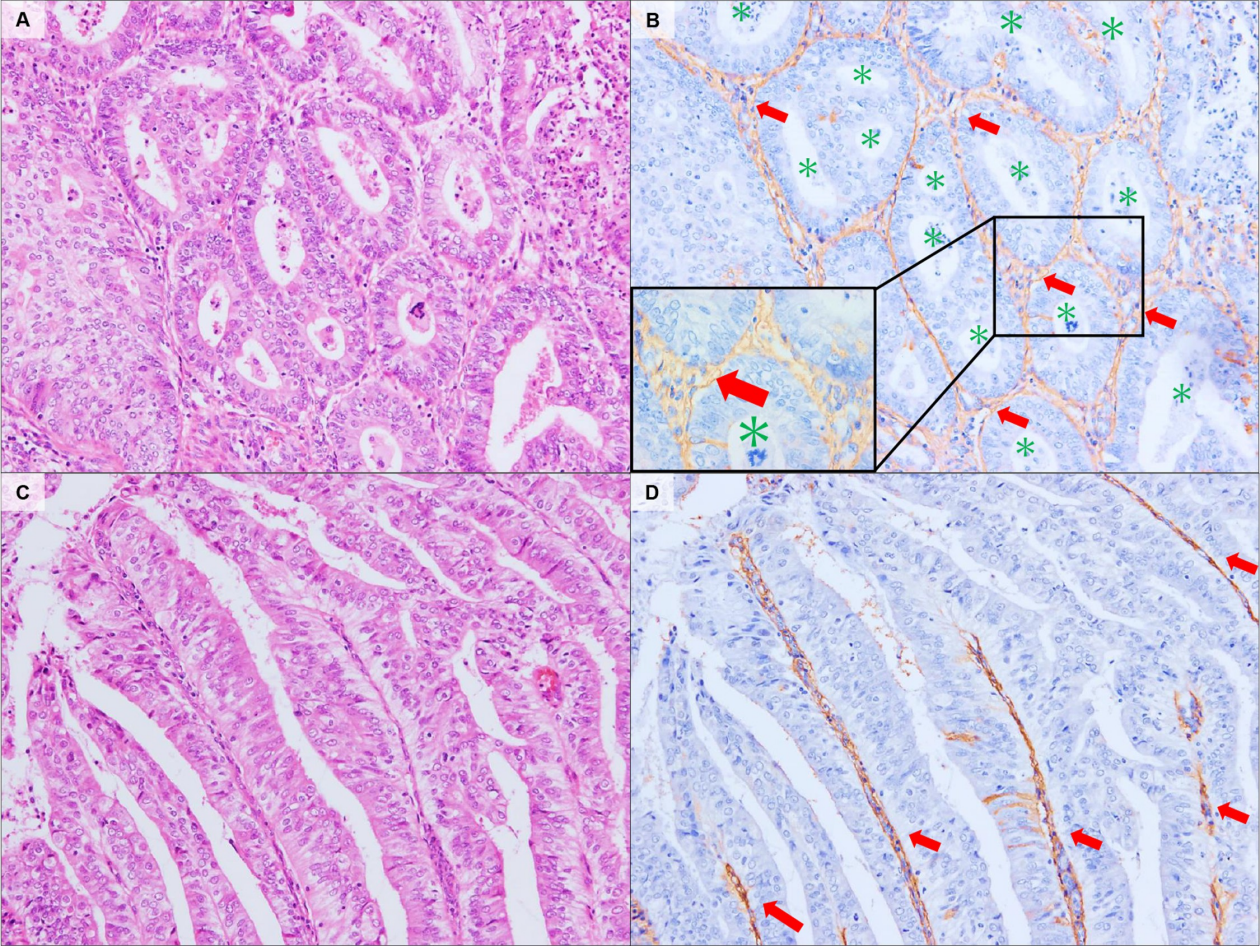
Representative stromal localization of Chondroitin Sulfate in G1-EEC. Back-to-back glandular crowding (A) and papillary architecture (C). CS (red arrows) showed reactivity surrounding the glandular lumen (green asterisk) and the fine fibrovascular stroma supporting the papillary architecture (B, D). Hematoxylin and eosin (H&E) staining results (A, C). CS-immunostaining (B, D). Original magnification: A–D, 100×.

**Fig 2:**
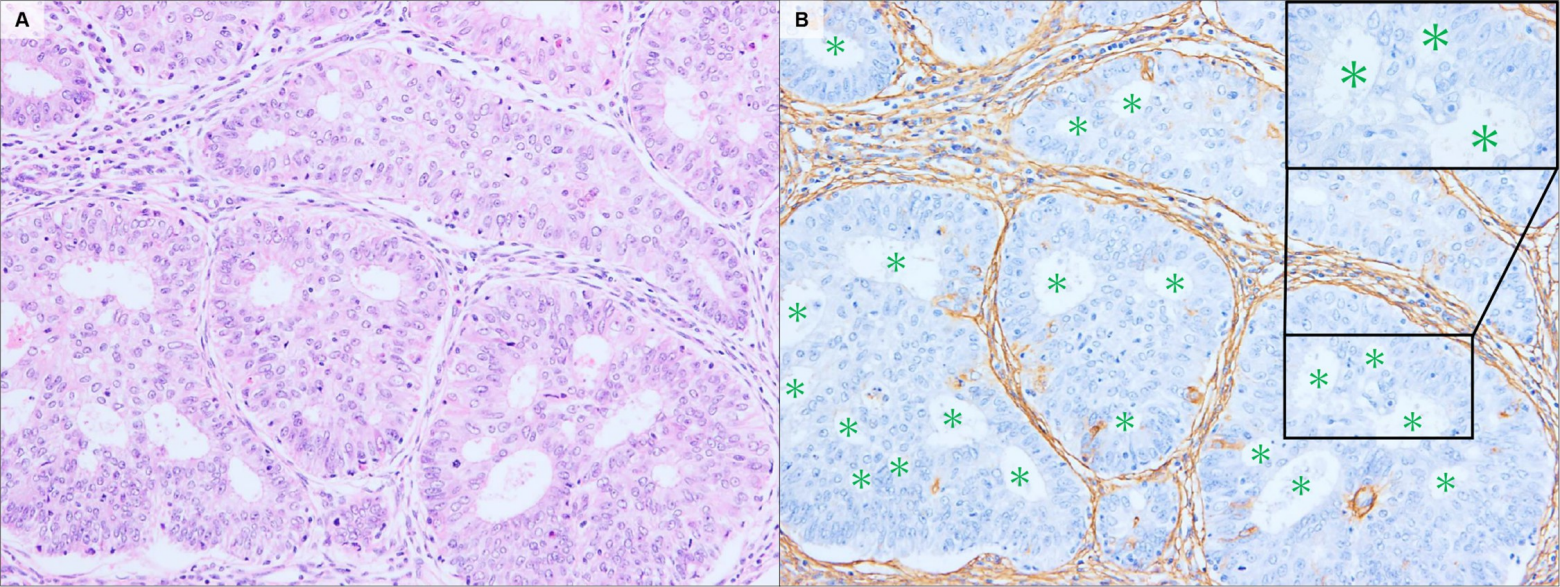
Representative stromal localization of CS in a cribriform pattern. Infiltrating fused tumor gland forming a glandular lumen without intervening stroma (A). CS localization reflects this architecture, and CS-immunostaining is poorly reactive around the glandular lumen (green asterisks) (B). H&E staining (A); CS-immunostaining (B). Original magnification: A–B, 100×.

**Fig 3:**
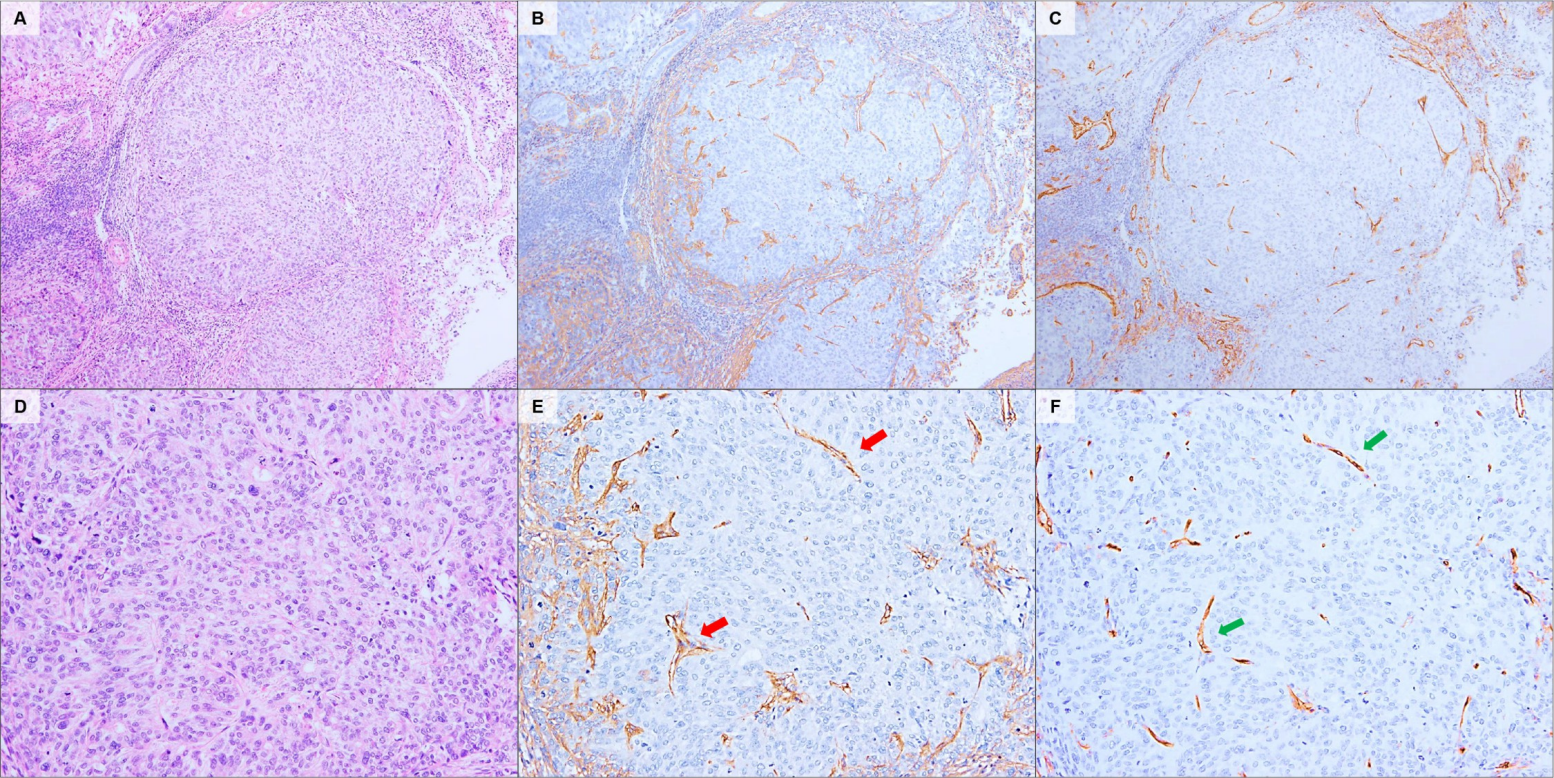
Representative stromal localization of CS in a solid growth pattern. In solid growth patterns such as G3-EEC (A, D), CS (B, E: red arrows) exhibited dot- or liner-like expression patterns in concordance with endothelial cells that expressed the CD31 marker (C, F: green arrows). H&E staining (A, D). CS-immunostaining (B, E). CD31-immunostaining (C.F). Original magnification: A–C, 40×; C–E, 100×.

Using immunostaining with a monoclonal antibody, we identified the localization of CS in various types of ECs. For the G1-EEC, CS showed reactivity with fibrovascular stroma, supporting closely-packed glandular crowding (back-to-back; Fig 1A and 1B) and papillary structures (Fig 1C and 1D). As the tumors became more invasive, the stromal structures were reconstructed. In the periglandular region, CS showed a similar staining intensity between G1-EEC (mean score, 2.10) and G2-EEC (mean score, 1.83; P3 = 0.114); however, the distribution of staining showed a significant difference (2.60 in G1-EEC vs in 1.50 in G2-EEC, P = 0.024). The difference in the total CS score between the two groups was statistically significant (4.70 in the G1-EEC vs 3.33 in the G2-EEC; P = 0.025; Table 1). This decrease in the distribution score indicated a lack of pre-existing endometrial stroma caused by tumor invasion and gland fusion (Fig 2). The number of compartments in which the tumor nest was separated by CS-positive stroma was significantly reduced: 8.12 for the G1-EEC, 4.83 for the G2-EEC, and 1.76 for the G3-EEC (P < 0.001 for each; S1 Fig). Inside the tumor glands with solid growth patterns, such as the G3-EEC, CS exhibited a dot- or liner-like expression pattern in concordance with the cells that expressed CD31—an endothelial cell marker (Fig 3).

**Table 1:** CS-immunostaining scores in the peri glandular stromal region.

	G1-EEC	G2-EEC	P-value
Score	Mean (SD)	Mean (SD)	
Intensity score	2.10 (0.30)	1.83 (0.37)	0.114
Distribution score	2.60 (0.66)	1.50 (0.76)	0.024*
Total score	4.70 (0.90)	3.33 (0.94)	0.025*

CS, chondroitin sulfate; G1-EEC, grade 1-endometrial endometrioid carcinoma; G2-EEC, grade 2-endometrial endometrioid carcinoma.

*Significant difference (P < 0.05).

**Significant difference (P < 0.01).

**Fig 4:**
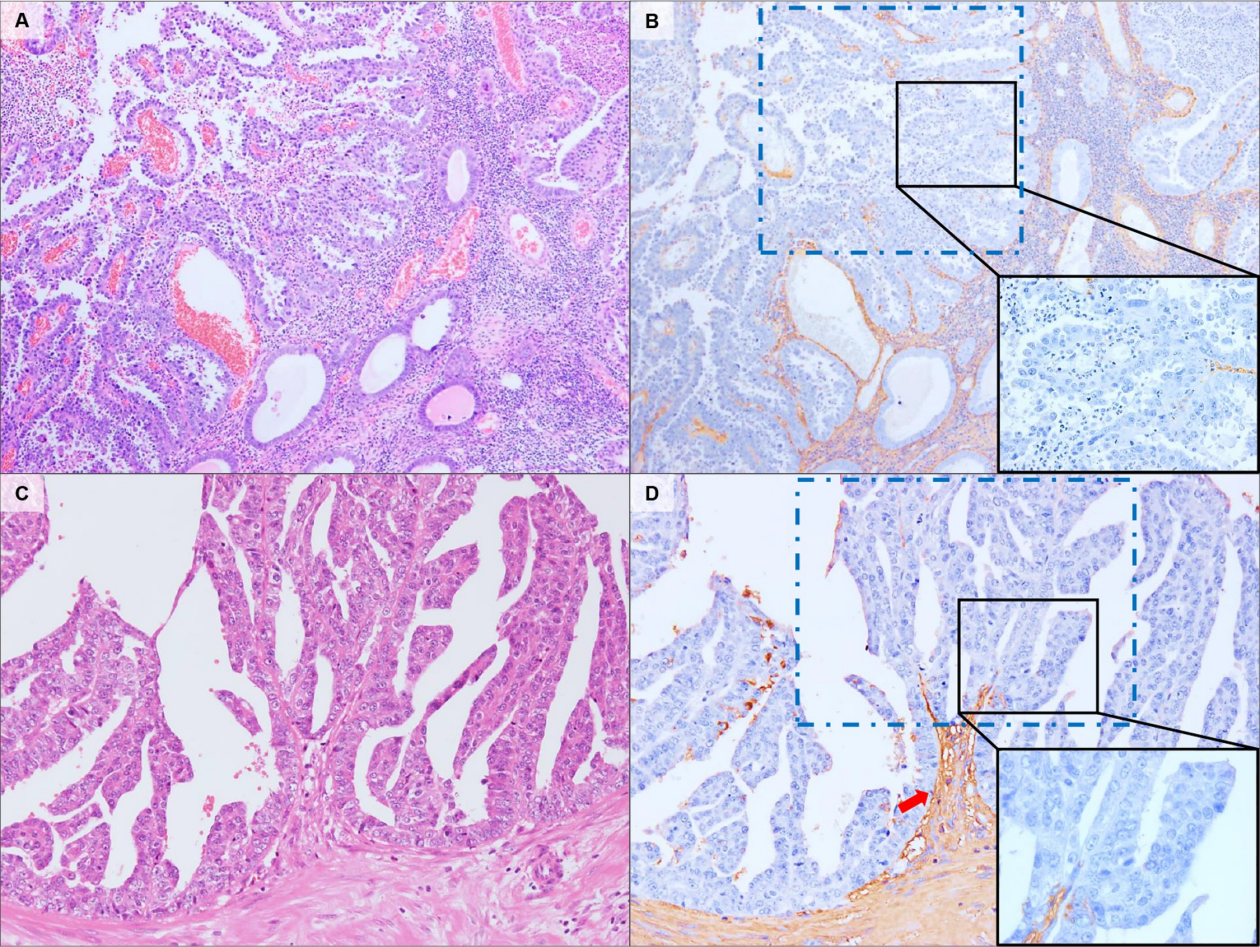
Representative stromal localization of CS in ESC. This tumor type showed a predominant papillary architecture with irregular papillae (A, C). The thick fibrous strands showed immunoreactivity via CS-immunostaining (red arrows) but poor reactivity in the elongated, branched, and delicate stromal regions (blue frame; B, D). H&E staining (A, C). CS-immunostaining (B, D). Original magnification: A–B, 40×; C–D, 100×.

In the ESC, thick fibrous strands supporting the papillary architecture showed reactivity with CS. By contrast, the area where the delicate stromal region branched into a narrow region showed poor reactivity with CS (Fig 4).

### Alcian Blue staining

**Fig 5:**
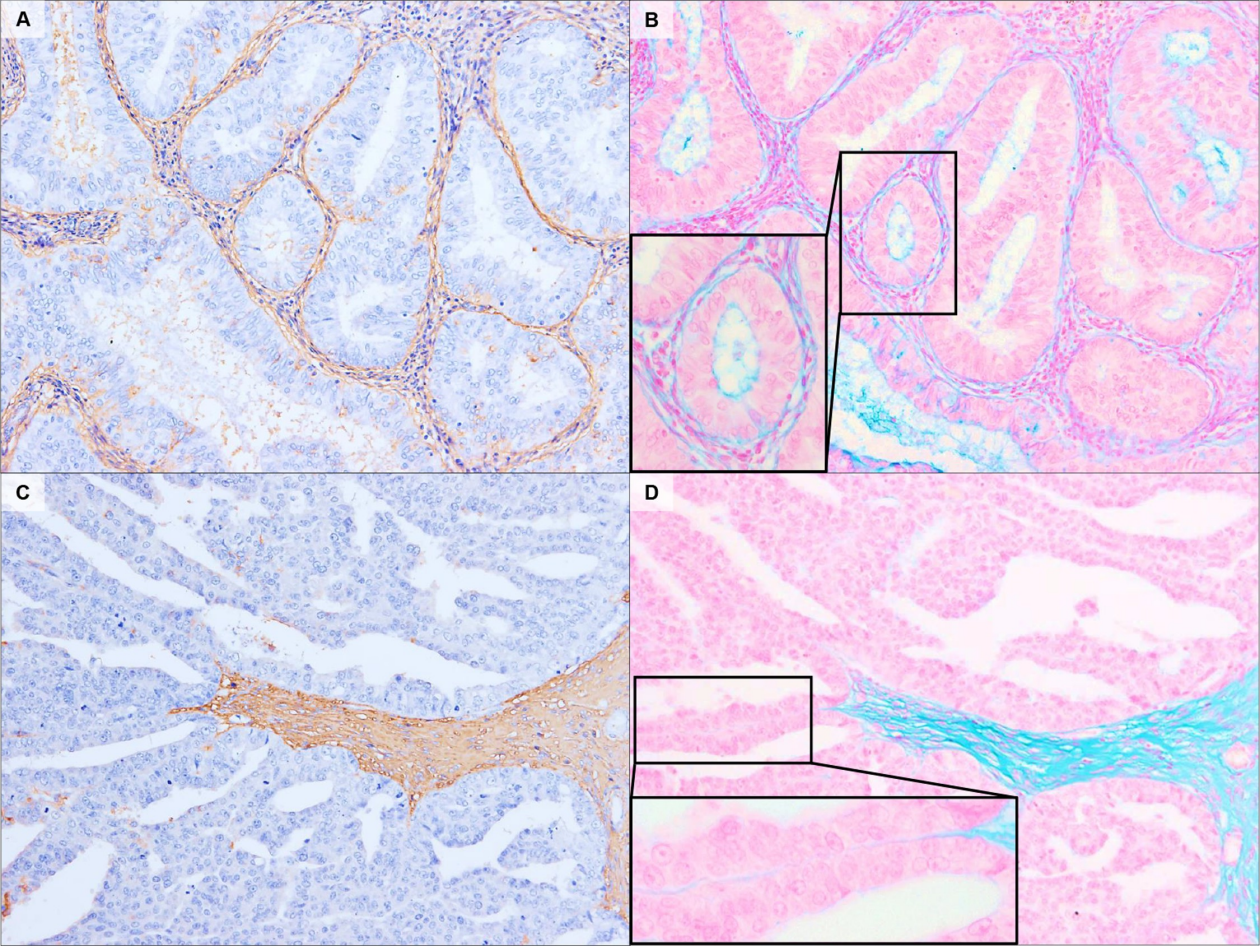
Typical AB staining patterns in EC. Representative immunohistochemical localization of CS in G1-EEC (A) and ESC (C). AB pH 1.0 staining showed similar reactivity with CS-immunostaining results (B, D). CS-immunostaining (A, C). AB staining (B, D). Original magnification: A–D, 100×.

AB staining for CS showed reactivity similar to that of immunostaining. It showed high reactivity with fibrovascular stroma, supporting dense glandular communities in the G1-EEC (Fig 5A and 5B). It showed poor reactivity with the stromal region, supporting narrowly branching, delicate papillary structures in the ESC samples (Fig 5C and 5D).

## Discussion

In this study, we assess the localization of CS, a primary ECM component of the endometrial stroma, in various types of ECs.

In G1-EEC, columnar tumor cells resembling the endometrial epithelium assume a tubular or papillary form. Findings that imply infiltration to the stroma include closely packed glandular patterns in the malignant glands, back-to-back glands with scanty stroma, and complex papillary growth patterns with blood vessels [13]. In this study, CS showed reactivity with these endometrial cancerous stromal regions, supporting the tumor tissue structure (Fig 1). As the grade increased, the original intervening stroma disappeared—owing to the invasion of excessively proliferated tumor cells—and the fibrous stroma supporting the tumor nest—such as dense cribriform and solid patterns—was reconstructed (Figs 2 and 3). The localization of CS reflected changes in these stromal structures, and a decrease in the number of tumor nests surrounded by CS-positive regions was observed (Table 1, and S1 Fig). In addition, CS showed reactivity with the delicate fibrovascular stroma buried within the tumor, as well as the endometrial cancerous stromal region surrounding the tumor nest (Fig 3). Thus, the localization of CS in the ECC clearly reflected changes in stromal structure.

ESC is a prototype of type II EC, which is estrogen-independent and exhibits a predominantly papillary structure with delicate fibrovascular stroma or thick fibrous strands—or, occasionally, tubular structures or slit-like spaces [14]. Notably, although the thick fibrous strands showed immunoreactivity with CS in this study, the delicate stromal region showed little or no reactivity with CS-immunostaining in the areas where it branched into narrow strands (Fig 4). These results suggest that the localization of CS in ESC behaves partly differently from stromal structures. In addition, ESC rarely shows a solid or pseudo-glandular pattern [15], which merits further scrutiny.

AB pH 1.0 staining is a conventional staining method that targets CS and other substances with sulfonic groups [16]. We have preliminarily confirmed that observation of the AB staining pattern of cell clusters in endometrial cytology specimens is useful in determining G1-EEC; however, histological analysis was not performed. The present study confirmed that AB staining corresponds to the CS localization of the stroma in EEC (Fig 5). In cytology, where three-dimensional cell clusters appear, stromal assessment is an important clue for cytologic examination [17]. In the future, a more detailed analysis comparing the staining of the corresponding tissue and cytology specimens at each lesion should be performed. Since AB staining does not require complex procedures such as peroxidase blocking or antibody-antigen reactions, it can contribute as a simple, rapid, and inexpensive stromal assessment tool.

For the classification of ECs, immunohistochemical markers, such as p53, p16, and IMP3, have been established [18–20]. However, assessment focused on the stromal region around tumor cells has not been adequately addressed. The present attempt could be applied to cytology and pathological exploration in other areas, such as the ovary, pancreas, and breast.

In this study, we assess the characteristic localization of CS in various EC types. The present study provides new information on endometrial stromal assessment.

## Supporting information

S1 Fig Tumor nests separated by CS-immunohistostaining. Consistent with the loss of intervening stroma as tumor grade increased, the number of tumor nests (green asterisk) surrounded by CS-positive stroma was significantly reduced. G1-EEC (A), G2-EEC (B), and G3-EEC (C). Steel-Dwass test analysis of between-grade differences in EEC (D). CS-immunohistostaining (A–C). ***P < 0.001. Circles represent outliers, and crosses represent averages.
(PDF)

S1 Table Evaluation of staining. All raw data on the staining score and tumor nest count are available in the Excel file.
(XLSX)
