# Supplementary material for: Assessment of the localization of chondroitin sulfate in various types of endometrial carcinoma
Source: PLoS One. 2024 May 28;19(5):e0304420. doi: 10.1371/journal.pone.0304420 (PMC11132439; doi:10.1371/journal.pone.0304420)
Supplement: S1 Fig — Consistent with the loss of intervening stroma as tumor grade increased, the number of tumor nests (green asterisk) surrounded by CS-positive stroma was significantly reduced. G1-EEC (A), G2-EEC (B), and G3-EEC (C). Steel-Dwass test analysis of between-grade differences in EEC (D). CS-immunohistostaining (A–C). ***P < 0.001. Circles represent outliers, and crosses represent averages. (PDF) [file pone.0304420.s001.pdf]

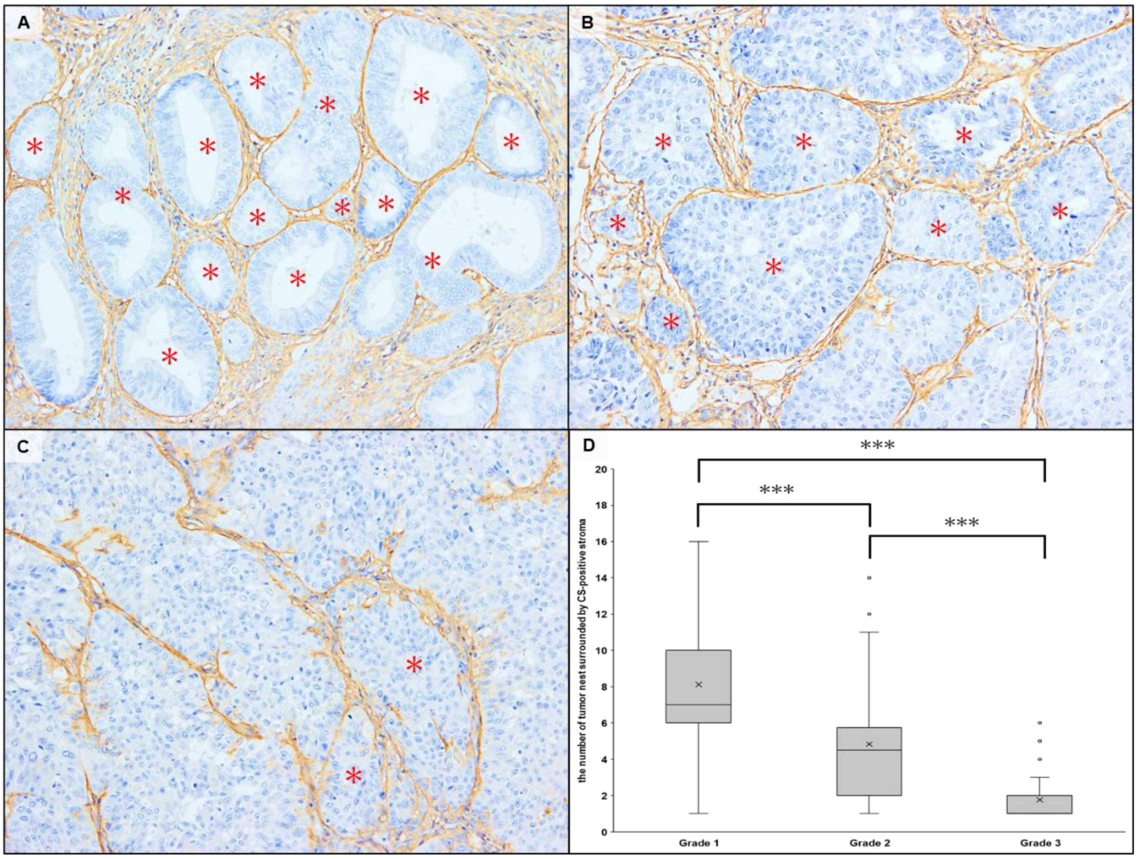

S1 Fig. Tumor nests separated by CS-immunohistostaining.

Consistent with the loss of intervening stroma as tumor grade increased, the number of tumor nests (green asterisk) surrounded by CS-positive stroma was significantly reduced. G1-EEC (A), G2-EEC (B), and G3-EEC (C). Steel-Dwass test analysis of between-grade differences in EEC (D). CS-immunohistostaining (A–C). ***$P < 0.001$. Circles represent outliers, and crosses represent averages.
